# Optimizing Mechanical Properties of Recycled 3D-Printed PLA Parts for Sustainable Packaging Solutions Using Experimental Analysis and Machine Learning

**DOI:** 10.3390/polym16233268

**Published:** 2024-11-24

**Authors:** Maria Tănase, Alexandra Ileana Portoacă, Alin Diniță, Gheorghe Brănoiu, Florin Zamfir, Elena-Emilia Sirbu, Cătălina Călin

**Affiliations:** 1 Mechanical Engineering Department, Petroleum-Gas University of Ploiești, 100680 Ploiesti, Romania; maria.tanase@upg-ploiesti.ro (M.T.); adinita@upg-ploiesti.ro (A.D.); 2 Petroleum Geology and Reservoir Engineering Department, Petroleum-Gas University of Ploiești, 100680 Ploiesti, Romania; gheorghe.branoiu@upg-ploiesti.ro; 3 Automation, Computers and Electronics Department, Petroleum-Gas University of Ploiești, 100680 Ploiesti, Romania; 4 Chemistry Department, Petroleum-Gas University of Ploiești, 100680 Ploiesti, Romania; elena.oprescu@upg-ploiesti.ro (E.-E.S.); catalina.calin20@yahoo.com (C.C.)

**Keywords:** PLA, recycling, 3D printing, XRD, DSC, mechanical performance, printing parameters, annealing, machine learning

## Abstract

Increasing environmental concerns and the need for sustainable materials have driven a focus towards the utilization of recycled polylactic acid (PLA) in additive manufacturing as PLA offers advantages over other thermoplastics, including biodegradability, ease of processing, and a lower environmental impact during production. This study explores the optimization of the mechanical properties of recycled PLA parts through a combination of experimental and machine learning approaches. A series of experiments were conducted to investigate the impact of various processing parameters, such as layer thickness and infill density, as well as annealing conditions, on the mechanical properties of recycled PLA parts. Machine learning algorithms have proven the possibility to predict tensile behavior with an average error of 6.059%. The results demonstrate that specific combinations of processing parameters and post-treatment annealing differently improve the mechanical properties (with 7.31% in ultimate tensile strength (UTS), 0.28% in Young’s modulus, and 3.68% in elongation) and crystallinity (with 22.33%) of recycled PLA according to XRD analysis, making it a viable alternative to virgin PLA in various applications such as sustainable packaging solutions, including biodegradable containers, clamshell packaging, and protective inserts. The optimized recycled PLA parts exhibited mechanical properties and crystallinity levels comparable to those of their virgin counterparts, highlighting their potential for reducing environmental impact and saving costs. For both as-built and annealed samples, the optimal settings for achieving high composite desirability involved a 0.2 mm layer thickness, with 75% infill for the as-built samples and 100% infill for the annealed samples. This study provides a comprehensive framework for optimizing recycled PLA in additive manufacturing, contributing to the advancement of sustainable material engineering and the circular economy.

## 1. Introduction

The growing demand for sustainable materials has led to an increased focus on recycling within the polymer industry, particularly for materials used in 3D printing [1,2,3,4,5]. Olawumi et al. [2] expanded their scope by exploring the conversion of various waste materials, including PLA and other polymers, into functional products. Their research underscores the importance of developing processing techniques that can enhance the mechanical properties of recycled materials, making them competitive with virgin materials. This work reinforces the idea that recycling and upcycling are not only environmentally beneficial but capable of producing materials with desirable mechanical properties. Maraveas et al. [6] evaluated the viability of recycled plastics, including PLA and other polymers, in 3D printing for industrial applications. Their research shows that the mechanical properties of recycled materials can be enhanced through blending with other polymers, and blending recycled plastics with other materials makes them suitable for 3D printing applications. For instance, Fused Deposition Modeling (FDM) can process plastic waste into durable filaments for 3D printing. E-waste plastics, such as polycarbonates, can be converted into filaments, while recycled PLA is often processed into industrial-grade filaments through extrusion and molding. Blending all these polymers like PET, PLA, and ABS is a specific mechanism of improvement of their mechanical characteristics. Additionally, recycled PET, commonly found in packaging, is repurposed into filaments for prototyping and other applications. Together, these methods illustrate how 3D printing is transforming plastic waste into valuable raw materials.

Additive manufacturing, another term for 3D printing, offers unique advantages in terms of design flexibility, material efficiency, and the ability to create complex geometries. However, the mechanical properties of 3D-printed parts, particularly those made from recycled materials, can vary significantly depending on factors such as the recycling process, material degradation, and the printing parameters used. This variability presents challenges in ensuring that recycled materials can be reliably used in applications where mechanical performance is critical. One method to improve the mechanical properties of recycled and upcycled thermoplastic materials is by adjusting the fiber orientation and weight fraction in thermoplastic composite filaments using the material extrusion filament process. Proper control over fiber alignment and loading can significantly enhance the strength, stiffness, and durability of printed parts. Recent studies, such as those on recycled glass fiber-reinforced polypropylene composites [7], the 3D printing of industrial waste polypropylene with carbon fiber reinforcement [8], and the upcycling of waste polypropylene with basalt fiber reinforcement [9], have demonstrated how fiber reinforcement during the material extrusion process can improve the mechanical performance of recycled and upcycled thermoplastic materials in additive manufacturing.

The drive towards sustainability and the reduction in plastic waste has propelled research into biodegradable and recyclable materials, with PLA being a prominent example due to its origin from renewable resources like corn starch and sugarcane. PLA’s application in 3D printing and other manufacturing sectors is favored for its biodegradability and commendable mechanical properties [10]. However, the recycling of PLA poses challenges, particularly in maintaining its mechanical integrity after multiple recycling cycles. The mechanical properties of recycled PLA are often inferior to those of virgin PLA due to thermal degradation, chain scission, and the introduction of impurities during the recycling process. For example, Hidalgo et al. [11] explored the reprocessing of 3D-printed PLA waste, comparing two types: a mixed waste blend from various PPE components and PLA waste from a single, known commercial source. Both materials were subjected to repeated extrusion cycles and processed into films via compression molding. Their results indicated that PLA from a known source withstands up to three extrusion cycles without significant degradation, whereas the mixed-waste blend degrades after two cycles. Their study highlights the importance of material traceability in recycling, proposing that known-source PLA offers better reprocessing potential compared to mixed-origin waste. Another study [12] focuses on the characterization and recycling of PLA waste from 3D printing. It examines two types of PLA waste, one from a known PLA grade and another from a mixed PLA waste collection, from corona makers in Madrid. Their research found that the PLA recycled from the known grade maintains properties similar to the virgin material, whereas the recycled material from the mixed PLA waste shows reduced viscosity, increased crystallization, and decreased transparency. These findings emphasize the importance of sorting and accurately characterizing 3D printing waste to achieve high-quality recycled materials.

Similarly, the work in [13] examines replacing virgin PLA with recycled PLA in additive manufacturing. Both types of PLA filaments were analyzed using IR spectroscopy, TGA, and dynamic rheology, then tested in 3D printing. The recycled PLA showed slightly reduced IR absorption, but similar thermal and mechanical properties compared to the virgin PLA. Both materials had a similar thermal degradation onset at 315 °C and comparable viscosity trends, with a 30% reduction after 10 min. Drying improved rheological stability, and the 3D-printed parts from both PLA types had no significant differences in their storage modulus or dissipation factor. Another study [14] investigated the recyclability of PLA in extrusion-based additive manufacturing by comparing blends of recycled PLA (rPLA) and virgin PLA to coextruded PLA filaments. Specifically, it evaluated whether coextrusion—where a high-rPLA core is paired with a virgin PLA skin—is more effective than blending. The findings revealed that while the strength of individual strands remained relatively consistent, layer adhesion decreased by up to 67%. Additionally, the coextruded filaments were found to be more brittle and exhibited poor weld lines between their layers, leading to reduced tensile strength. Nevertheless, the coextruded filaments offer economic advantages, as they save on master batching and colorants due to the outer layer’s significant impact on the part’s coloring. Bergaliyeva et al. [15] investigated the thermal, morphological, and mechanical properties of 3D-printed PLA blends with varying ratios of virgin and recycled material (100/0, 25/75, 50/50, 75/25). The recycled PLA, derived from shredded real waste, showed minimal impact on thermal stability, with both virgin and recycled PLA degrading similarly. A higher recycled content reduced crystallinity and resulted in a more heterogeneous structure. Interestingly, tensile strength improved with more recycled PLA, from 44.20 MPa for pure PLA to 52.61 MPa for the blend with the highest recycled content, though reprocessed parts exhibited distinct mechanical properties.

However, PLA is just one of many materials being recycled and reused in 3D printing. The recycling of thermoplastics like Acrylonitrile Butadiene Styrene (ABS) [16,17], polypropylene (PP) [18,19,20], and polyethylene (PE) [21,22,23,24,25], among others, is also important for reducing environmental impact. Each of these materials presents its own set of challenges and opportunities when it comes to recycling and mechanical property optimization.

The present work introduces an approach to enhancing the mechanical properties of recycled PLA, a widely used biodegradable polymer in 3D printing. This study’s innovation lies in its combination of experimental techniques and machine learning algorithms to systematically optimize the processing parameters and post-processing treatments that influence the mechanical performance of recycled PLA. This dual approach not only advances the understanding of how specific factors affect the material properties but provides a predictive framework for achieving near-virgin PLA quality in recycled parts. The practical implications of this research are significant; it supports the broader adoption of recycled PLA in various industrial applications by demonstrating that with optimal processing, recycled PLA can achieve mechanical properties comparable to those of virgin PLA. This contributes to sustainable manufacturing practices by reducing reliance on virgin materials and minimizing environmental impact.

## 2. Bibliometric Analysis

**Figure 1:**
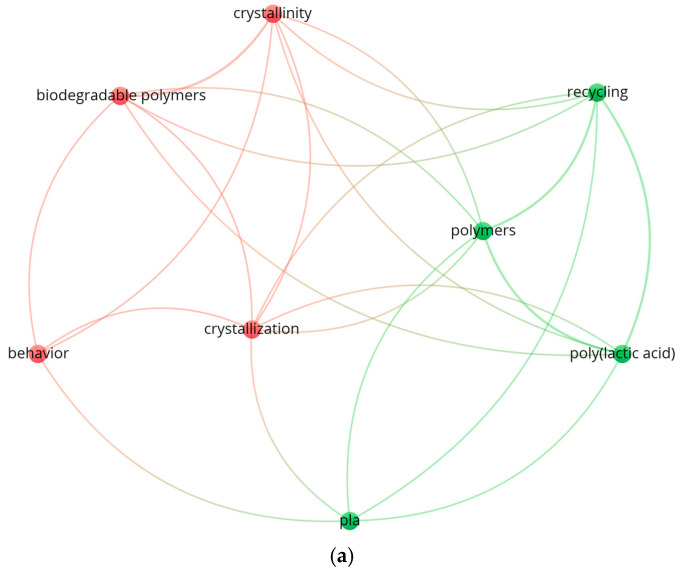

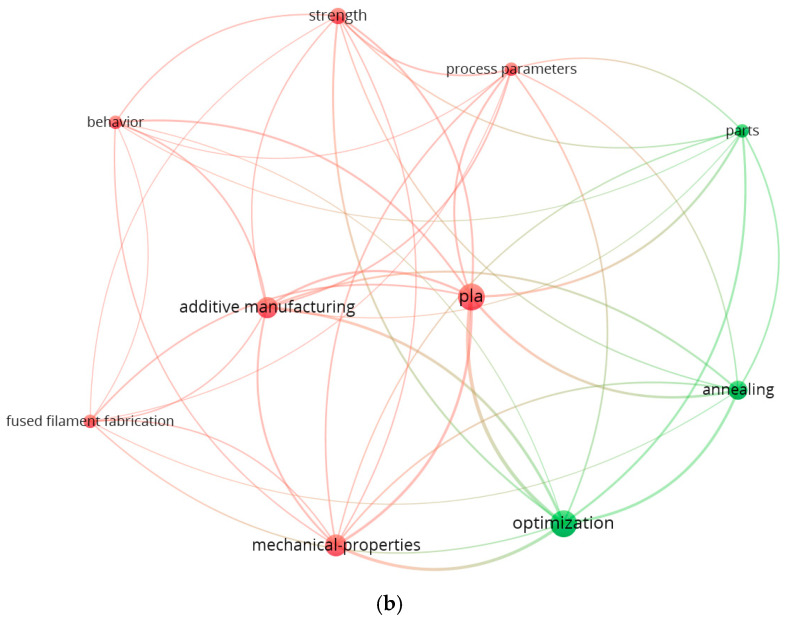
Co-occurrence analysis of author keywords: (**a**) WOS search using the terms (TS = (Recycled PLA)) OR TS = (Recycled polylactic acid) AND (TS = (annealing)) OR TS = (post-processing treatment); (**b**) WOS search using the terms TS = (3D-printed PLA)) AND (TS = (annealing)) OR TS = (post-processing treatment) AND (TS = (machine learning)) OR TS = (optimization).

A comprehensive search was conducted in the Web of Science (WOS) database to assess the current state of research related to recycled PLA and various enhancement techniques, as well as the application of machine learning for optimizing mechanical properties. The initial search query was formulated as (TS = (Recycled PLA)) OR TS = (Recycled polylactic acid) AND (TS = (annealing)) OR TS = (post-processing treatment) AND (TS = (machine learning)) OR TS = (optimization). This query aimed to identify studies that connected recycled PLA with both post-processing treatments, such as annealing, and advanced analytical techniques, like machine learning or optimization. However, no results were found for this combined search, indicating a significant gap in the literature that combines these aspects. To further probe this gap, a simplified search was conducted using the terms (TS = (Recycled PLA)) OR TS = (Recycled polylactic acid) AND (TS = (annealing)) OR TS = (post-processing treatment). This yielded only five relevant sources, suggesting that while some research has been conducted on recycled PLA and post-processing treatments like annealing, the integration of advanced optimization methods (e.g., machine learning) into these treatments remains minimal. This finding supports the novelty of the present study, as it addresses this underexplored combination of approaches. The keyword ‘co-occurrence analysis’ shown in Figure 1a was searched with a minimum keyword occurrence set to two. Out of 43 keywords, only 8 met this threshold.

An additional search was conducted to explore the body of literature specifically on 3D-printed PLA in conjunction with annealing, post-processing treatments, and the use of advanced techniques like machine learning or optimization. The search query used was (TS = (3D-printed PLA)) AND (TS = (annealing)) OR TS = (post-processing treatment) AND (TS = (machine learning)) OR TS = (optimization).

This search returned only 36 sources, indicating that while there is a moderate amount of research involving 3D-printed PLA and post-processing treatments such as annealing, the intersection with machine learning or optimization techniques remains limited. This result highlights that the application of machine learning or systematic optimization in enhancing the properties of 3D-printed PLA through post-processing treatments is not widely studied. The keyword ‘co-occurrence analysis’ depicted in Figure 1b was searched with a minimum keyword occurrence of five. Among 205 keywords, only 15 met this threshold.

These findings collectively underscore the novelty of the current study. By focusing on a combination of experimental techniques and machine learning to optimize the mechanical properties of recycled and 3D-printed PLA, this research fills a critical gap in the literature where advanced analytical tools have not been commonly integrated with post-processing treatments for recycled materials.

## 3. Materials and Methods

### 3.1. Design of Experiments and Sample Fabrication

**Figure 2:**
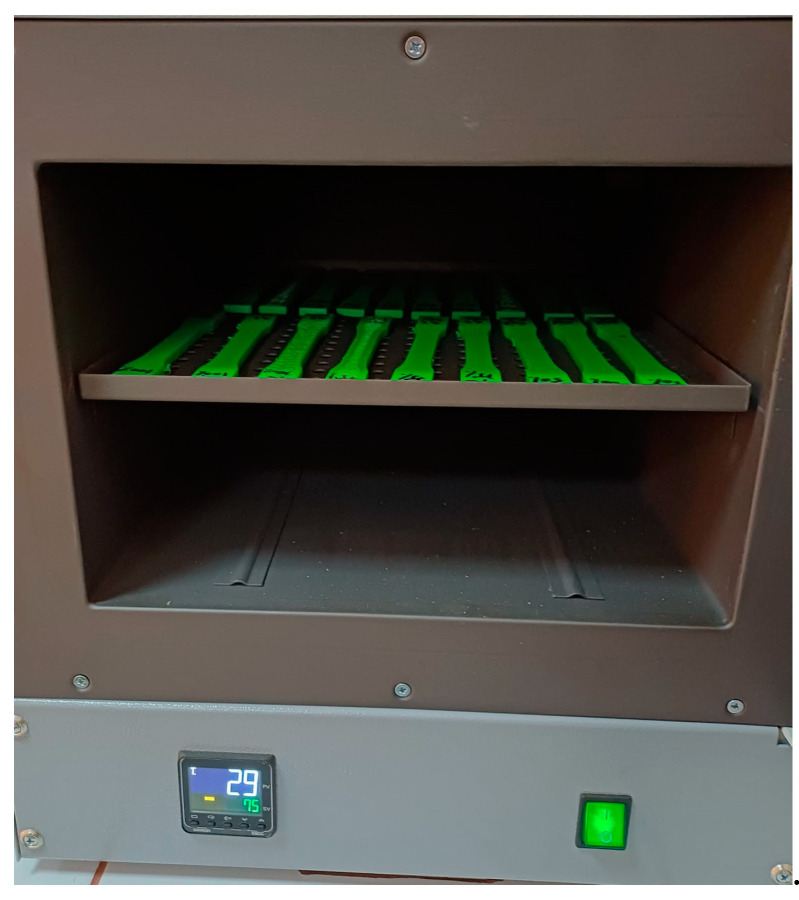
Three-dimensionally printed recycled PLA samples subjected to annealing treatment.

In this experimental study, three infill percentages (50%, 75%, and 100%) and three layer thicknesses (0.10 mm, 0.15 mm, and 0.20 mm) were considered as variable parameters for the 3D-printed samples to evaluate their mechanical characteristics and to facilitate a comparative analysis of the tensile properties of recycled PLA samples with those previously studied by the authors [26,27]. Tensile tests were conducted on both annealed and unannealed samples, and the results were compared. The annealing heat treatment involved subjecting the samples to a temperature of 75 °C for 3 h (Figure 2), and was performed using an oven with a controlled heating rate of 1 °C per second. Cooling was conducted gradually within the oven to ensure a uniform temperature decrease.

The specific printing settings (see Table 1) included an X–Y build orientation, model lines, and a 45° orientation.

The recycled filament (green color) was a known commercial PLA [28] (post-industrial), having, according to manufacturer, a density of 1.24 g/cm^3^ and glass transition temperature of 60.9 °C. The recycled PLA filament had not undergone any further processing prior to our analysis.

The full factorial design method was employed using Minitab 19 software to optimize the mechanical performance of 3D-printed recycled PLA parts. The analysis focused on two input parameters—infill percentage and layer thickness—each tested at three levels, as shown in Table 2.

The total number of experiments required is calculated based on the number of input factors (n) and levels (k), resulting in an orthogonal array of 9 tests, both for as-built and annealed samples. For each combination of printing parameters, 3 samples were printed and mechanically tested, resulting a total number of 54 3D-printed samples (27 samples as built and 27 samples subjected to annealing post-heat treatment).

To optimize the 3D printing process and improve the tensile properties of the printed materials, a systematic Design of Experiments (DOE) approach was applied. The main goal was to enhance their tensile properties: ultimate tensile strength (UTS), Young’s modulus (E), and elongation at break (A).

Statistical analysis, including Analysis of Variance (ANOVA), was used to identify the factors with the most significant impact. This step helped determine which variable—layer thickness or infill percentage—most strongly affected the tensile properties of the 3D-printed recycled PLA samples.

After identifying the critical factors, a desirability analysis optimization technique was employed to find the ideal combination of layer thickness and infill percentage that would maximize these tensile properties.

### 3.2. DSC Analysis

Differential scanning calorimetric (DSC) research was performed with a DSC 3+ Star system from METTLER TOLEDO (Leicester, UK), and the crystallization behavior of the material was examined in a N_2_ environment at 10 °C/min between 10 °C and 250 °C. The endothermic and exothermic peaks in the DSC thermograms were analyzed as part of the examination of the melting and crystallization properties of the studied material. The following Equation was used to calculate the degree of crystallinity (*X_C_*) based on the data obtained from the DSC thermogram:

(1) $$X_{C}=\frac{\Delta H_{m}}{w\cdot \Delta H_{m}^{100\%}}\cdot 100\left[\%\right]$$

where ∆*H_m_* is the measured melting enthalpy of PLA specimen [J/g], $\Delta H_{m}^{100\%}$ is the melting enthalpy for 100% crystalline PLA (93.7 J/g [29,30]), and *w* is the mass fraction of PLA in the analyzed sample.

In order to establish the annealing temperature for the 3D-printed samples, a DSC analysis was performed for the PLA recycled filament, and the glass transition temperature *T_g_* was obtained as 63.3 °C. The glass transition values were extracted from the DSC data by integration of the first peak and then the use of the determination glass transition temperature function of the device’s software.

### 3.3. XRD Analysis

The degree of structural order (crystallinity) of the PLA samples was investigated using an X-ray diffractometer D8 Advance (Bruker-AXS, Karlsruhe, Germany) with Cu-Kα radiation (λ = 1.54 Å), a Ni filter, a θ-θ configuration, and Bragg–Brentano geometry. The XRD measurements were applied on the PLA samples in the following scanning conditions: 40 KV, 40 mA, step 0.1°, scan speed 0.1°/5 s, and scanning angle (2θ) in the range 10–70°. The raw files were obtained using the XRD Commander program. Diffracplus EVA v14 software and the PDF-ICDD database were used for phase identification, and the Rietveld refinements were run in the Diffracplus TOPAS 4.1 software.

For XRD analysis, Equation (2) was used for determining the degree of crystallinity *X_C_*:

(2) $$X_{c}=\frac{\sum A_{crys}}{\sum A_{crys}+A_{amorph}}$$

where *A_crys_* represents the fitted areas of the crystal phase, and *A_amorph_* represents the fitted areas of the amorphous phase [31].

### 3.4. Tensile Testing

Eighteen groups of specimens, each consisting of three samples, were subjected to mechanical testing for determining their key material properties, including ultimate tensile strength (UTS), elongation at break (A), and tensile Young’s modulus (E). The tensile tests were performed according to the ASTM D638 standard [32] using an electro-mechanical machine equipped with a 2.5 kN load cell, operating at a speed of 5 mm/min, while elongation at break was measured using an axial extensometer.

### 3.5. Machine Learning Algorithms

When focusing on the tensile behavior of recycled PLA specifically in the context of 3D printing, machine learning is used to optimize various aspects of the printing process and material properties. Here are some tailored considerations and algorithms relevant to this area:

- Linear Regression is an algorithm that predicts a continuous outcome based on input variables. It assumes a linear relationship between the independent variables and the dependent variable, suggesting that changes in the inputs result in proportional changes in the output [33].
- Decision Tree is an algorithm that divides data into branches based on input characteristics. The data are separated into groups with similar labels and values [34].
- Random Forest is an algorithm that is based on the construction of a decision forest, using the Decision Tree algorithm. It aims to run the Decision Tree algorithm individually and choose the average values for the regressions [34].
- Gradient Boosting works similarly to Random Forest, only it creates sequential trees. Using this approach, each newly created tree will repair the errors of the previous ones [1].
- AdaBoost (Adaptive Boosting) is a ML algorithm that primarily enhances the performance of multiple weak learners, often decision stumps, by concentrating on challenging cases. These models are developed in sequence to address and rectify previous errors. The final model is formed as a weighted sum, giving priority to models that perform better [33].
- A Neural Network is a machine learning model mimicking the human brain’s structure, with interconnected layers of nodes (neurons) that learn patterns from data. It consists of neurons (nodes), layers (input layer, hidden layers, output layer), weights and biases, and activation functions [35].
- Support Vector Machine (SVM) is effective in high-dimensional spaces, particularly for tasks with complex class boundaries. Its goal is to find the optimal hyperplane that separates data points of different classes. The hyperplane acts as a decision boundary, dividing the space into regions for each class [35].
- The k-Nearest Neighbors (kNN) algorithm is a supervised ML technique. It predicts outcomes by locating the nearest data points (neighbors) to a query point, utilizing majority labels for classification or calculating average values for regression. As an instance-based or lazy learning method, kNN retains the training data without forming a model, making predictions based on the distance between data points [36].

The most used metrics in machine learning have been highlighted in numerous studies. The paper in [37] presents some of these metrics: Mean Square Error (MSE), Root Mean Square Error (RMSE), Mean Absolute Error (MAE), Mean Absolute Percentage Error (MAPE), and R-Squared. Mean Square Error measures the average of the squares of the errors, which is the average-squared difference between the predicted and actual values. Root Mean Squared Error provides a measure of error in the same units as the original data, making it easier to interpret. Mean Absolute Error evaluates the average of the absolute differences among predicted and actual values. Mean Absolute Percentage Error expresses the error as a percentage of the actual values, providing a relative degree of accuracy. R-squared indicates the proportion of the variance in the dependent variable that can be predicted from the independent variables, with higher values suggesting that the model explains a greater portion of the variance.

## 4. Results and Discussion

### 4.1. Mechanical Properties

**Figure 3:**
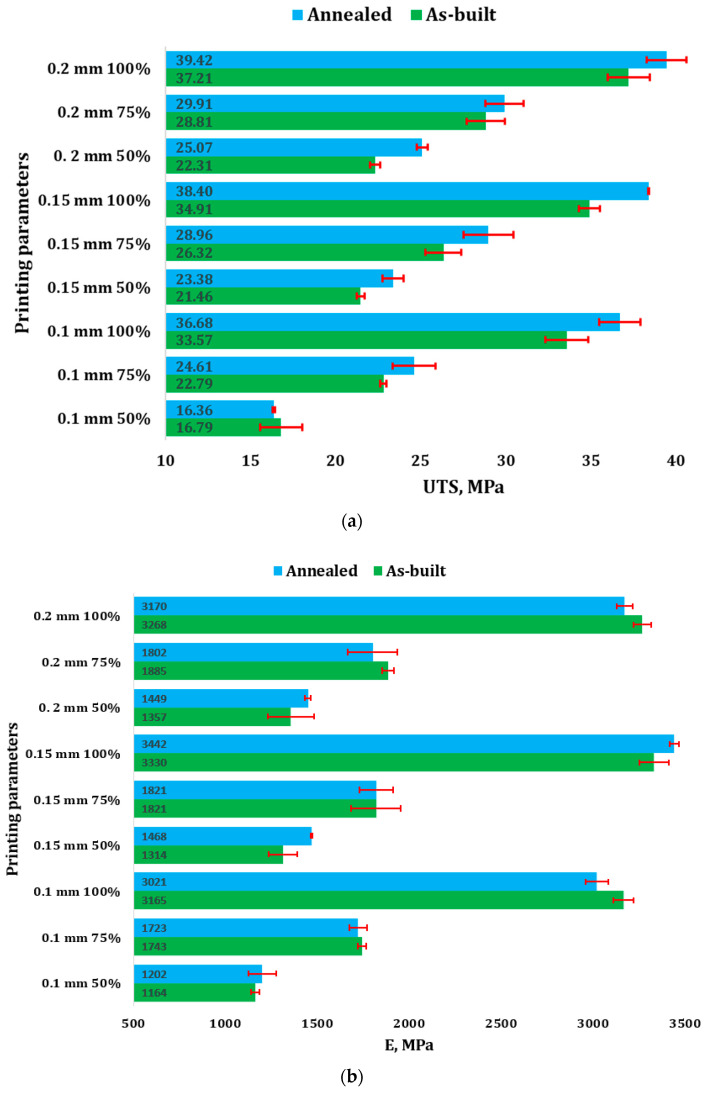

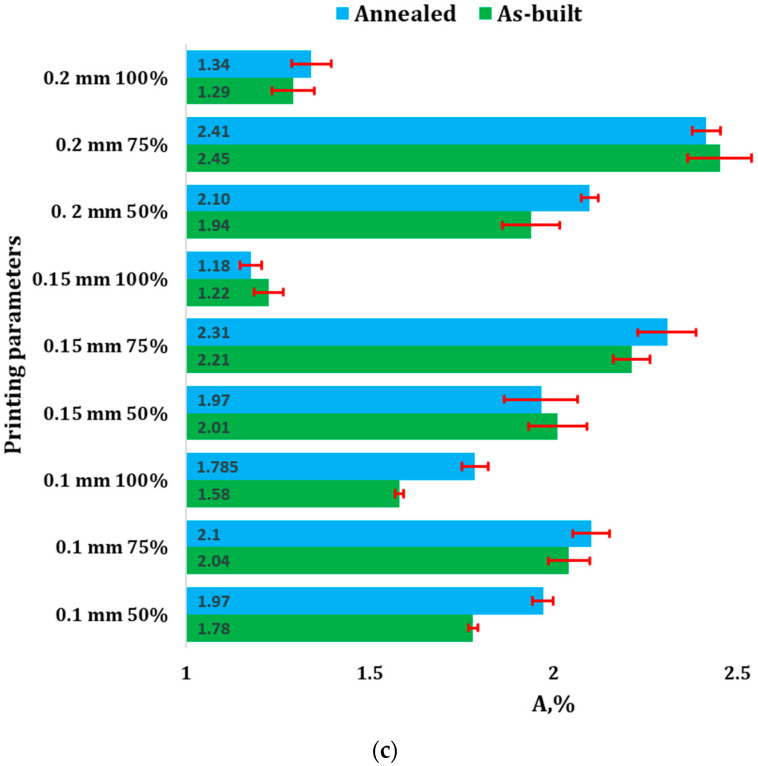
Mechanical properties of 3D-printed recycled PLA samples: (**a**) ultimate tensile strength (UTS); (**b**) Young’s Modulus; (**c**) elongation at break (A).

Figure 3 presents the comparative results regarding the tensile properties of both the investigated as-built and annealed 3D-printed recycled PLA samples.

It can be seen that for the as-built samples, the UTS increases with both layer thickness and infill density. For instance, at a 0.1 mm layer height and 50% infill, the UTS is 16.79 MPa, while at 100% infill, it jumps to 33.57 MPa. The same trend is seen for other thicknesses. Annealing generally improves the UTS slightly, especially at 100% infill (e.g., for 0.1 mm, UTS rises from 33.57 MPa to 36.68 MPa after annealing).

The Young’s modulus for the as-built samples increases with both infill density and layer height. For example, for the 0.1 mm layer at 100% infill, it is 3165 MPa, while for 50% infill, it is significantly lower at 1164 MPa. Annealing improves stiffness (E) slightly across certain conditions like at a 0.2 mm layer thickness and 50% infill, 0.15 mm layer thickness and 100% infill, 0.15 mm layer thickness and 50% infill, or 0.1 mm layer thickness and 50% infill, indicating better resistance to deformation after treatment.

Generally, for the as-built samples, elongation at break A decreased with increasing infill density, indicating less ductility at higher densities. For instance, at a 0.1 mm layer thickness and 100% infill, elongation drops to 1.58% from 1.78% at 50%. The elongation values show minor variations post-annealing, but the effect on ductility is generally less pronounced. For example, at 0.1 mm 50%, *A* slightly increases after annealing (from 1.78% to 1.97%).

The influence of annealing treatment on the tensile properties of 3D-printed PLA parts was investigated in [27], and it was found that the UTS of the annealed samples increased by 15%–36%, while in this case, for recycled PLA, the improvement in UTS was between 3.8% and 12.35%. As depicted in Figure 3a, for each set of parameters, the annealed samples consistently demonstrate higher UTS values compared to their as-built counterparts, indicating that the annealing process effectively enhances the material’s strength. This improvement is particularly noticeable in samples printed with a larger layer thickness (0.2 mm) and higher infill percentages (100% and 75%), where the difference between the annealed and as-built UTS values is most pronounced. The results suggest that annealing positively influences the material’s mechanical performance, likely due to increased molecular alignment or reduced internal stresses achieved through the heat treatment process.

Comparing with the results from [27], where the same printing parameters were considered for PLA 3D printing, when analyzing the effects of layer thickness on the strength of recycled versus virgin PLA, it is important to note that thicker layers (e.g., 0.2 mm) typically result in stronger prints compared to thinner layers. As such, the UTS ratios close to or above 1 observed for thicker layers (0.2 mm) indicate that recycled PLA can maintain or even exceed the strength of virgin PLA when using thicker layers. In contrast, thinner layers (e.g., 0.1 mm), while providing finer surface finishes, often reduce the bond strength between layers. This can lead to weaker printed parts because the thin layers are more prone to failure along the interlayer boundaries. Consequently, the lower UTS ratios for recycled PLA at 0.1 mm, especially at lower infill percentages, suggest a drop in tensile strength compared to virgin PLA under these conditions. The weakest performance is observed in the combination of thin layers and low infill, where the mechanical properties of recycled PLA are less favorable. Also, annealing enhances mechanical properties more effectively in specimens with a thicker layer thickness (0.2 mm) compared to those with a thinner layer thickness (0.1 mm). For 0.2 mm layers, the ultimate tensile strength (UTS) and Young’s modulus (E) show notable increases, especially at higher infill densities, suggesting that thicker layers allow for better molecular diffusion and interlayer bonding during annealing. In contrast, 0.1 mm layers see less improvement in UTS and stiffness, possibly due to the smaller cross-sectional area limiting structural reinforcement. Similarly, elongation at break (A%) improves slightly more for 0.2 mm layers, indicating increased ductility, whereas the effect is less consistent for 0.1 mm layers. Overall, thicker layers benefit more from annealing in terms of strength, stiffness, and ductility.

The effect of infill percentage becomes clearer when we account for these layer thickness differences. At higher infill percentages (e.g., 100%), the denser internal structure can compensate for some of the weaknesses caused by thinner layers. For instance, at 0.1 mm with 100% infill, the UTS ratio exceeds 1, indicating that a fully solid part can restore strength, even in thinner layers, allowing recycled PLA to perform similarly or slightly better than virgin PLA. However, at 50% infill, the combination of thin layers and reduced material density results in the most significant drop in tensile strength, as reflected by the UTS ratio of 0.664 for recycled PLA. This suggests that recycled PLA is more sensitive to lower infill percentages, particularly when paired with thinner layers. On the other hand, thicker layers (e.g., 0.2 mm) exhibit more consistent UTS ratios across different infill percentages. Even with lower infill values, recycled PLA at 0.2 mm maintains strength levels close to those of virgin PLA, with UTS ratios slightly above 1 [38]. This indicates that thicker layers provide a more robust structure, even when the internal density is reduced. The enhanced layer bonding in thicker layers ensures better overall strength retention in recycled PLA.

### 4.2. Machine Learning Predictions on Tensile Behavior

#### 4.2.1. Data Validation

For the implementation of the prediction algorithm, a dataset consisting of 35 tests was used. The dataset has the structure presented in Table 3. To improve the robustness of the prediction algorithm, experiments were conducted considering a wide range of scenarios. Table 4 reveals this by presenting some statistics of the dataset.

This step is important considering several aspects: Feature Correlation, Outlier Detection, and Distribution Analysis. Strong links between the features and targets will enhance success rates, and an investigation of outliers is necessary to identify the cause of the errors. Distribution Analysis is essential to reveal whether the data are focused or not.

#### 4.2.2. Data Visualization

**Figure 4:**
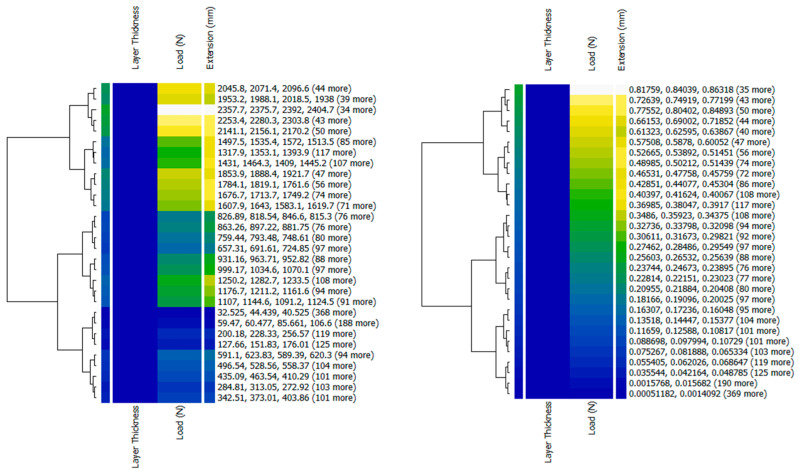
The intensity of data points illustrated with heatmaps.

In this section, various tools have been used to visualize the data (line graphs, scatter plots, and heatmaps). To achieve a data correlation, a series of visualizations are necessary. In Figure 4, heatmaps are used to illustrate the intensity of the data points across two dimensions. In this situation, the two numeric values Load and Extension were combined with categorical values. Clusters were used to view the data in a compressed form.

**Figure 5:**
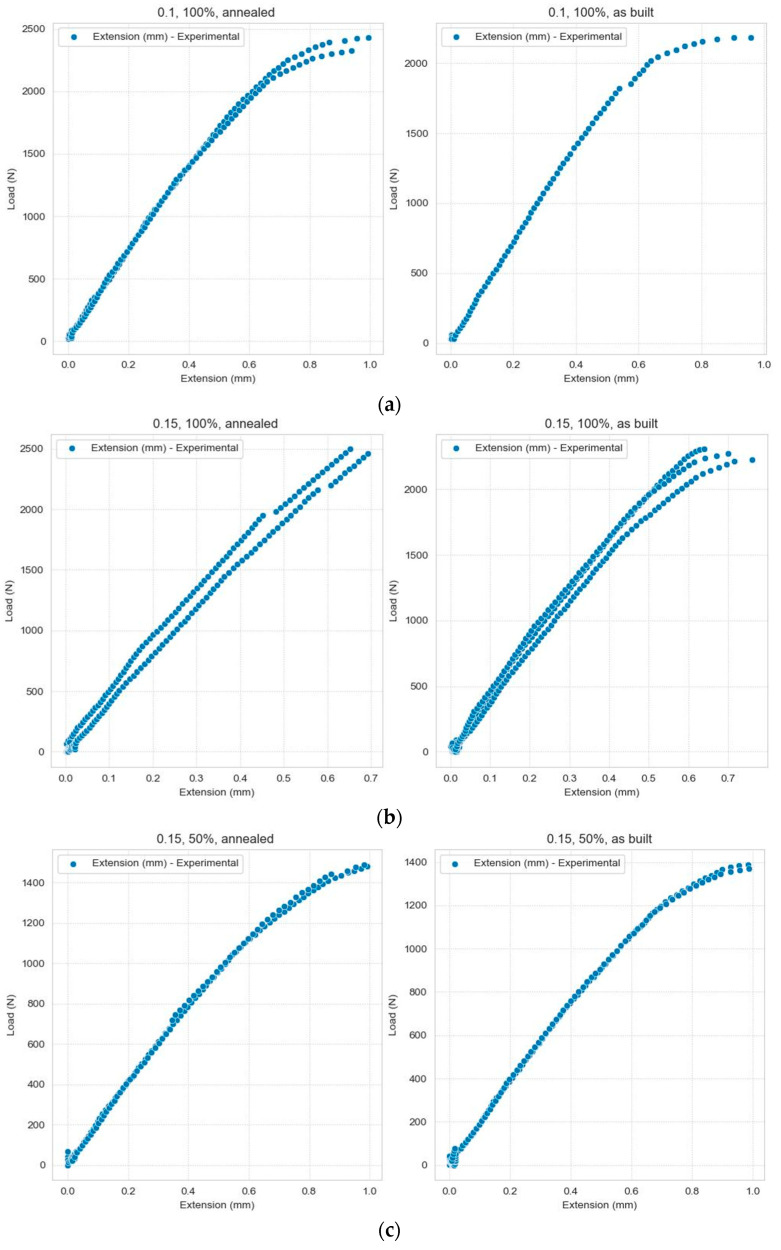

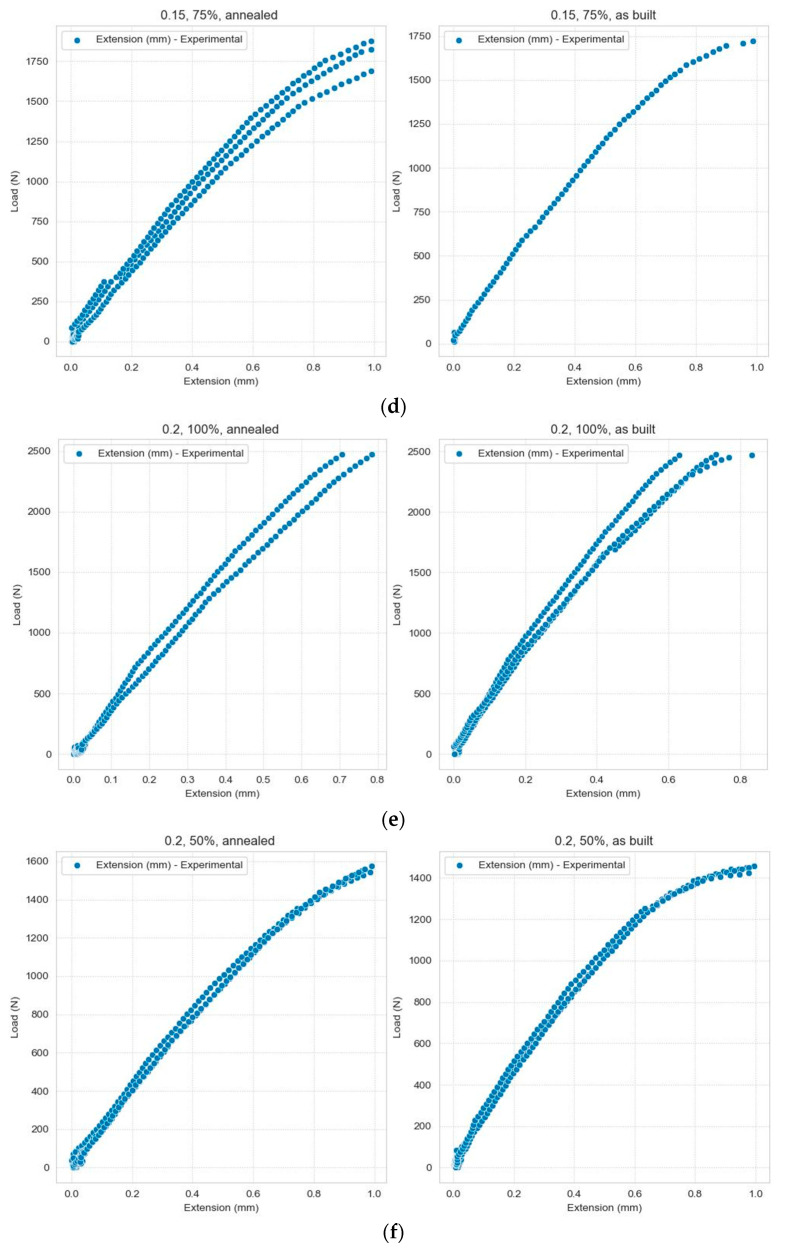

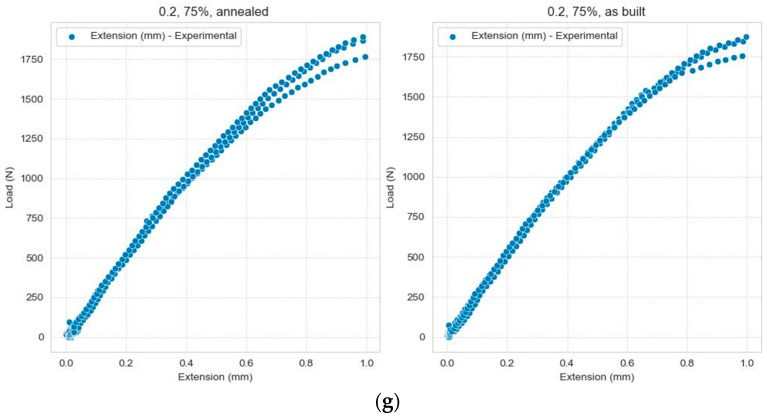
Variations in the Extension depending on the Load, grouped according to the three types of categorical data: (**a**) Layer Thickness = 0.1 mm, Infill density = 100%; (**b**) Layer Thickness = 0.15 mm, Infill density = 100%; (**c**) Layer Thickness = 0.15 mm, Infill density = 50%; (**d**) Layer Thickness = 0.15 mm, Infill density = 75%; (**e**) Layer Thickness = 0.2 mm, Infill density = 100%; (**f**) Layer Thickness = 0.2 mm, Infill density = 50%; (**g**) Layer Thickness = 0.2 mm, Infill density = 75%.

Heatmaps are not the only tools to see the distribution of values in the dataset. In Figure 5, a scatter plot is used to show variations in the Extension based on the Load, categorized by three types of categorical data: layer thickness, infill density, and samples.

**Figure 6:**
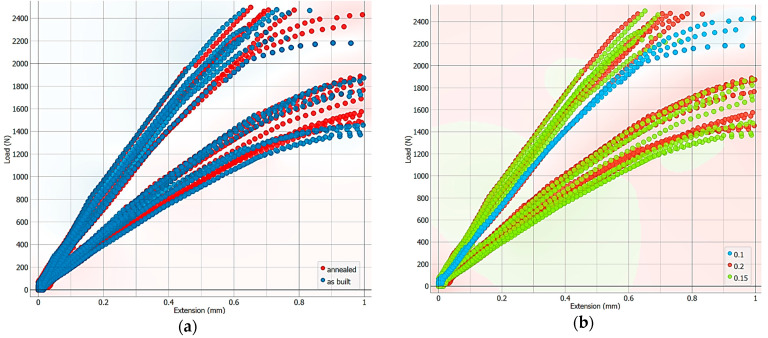

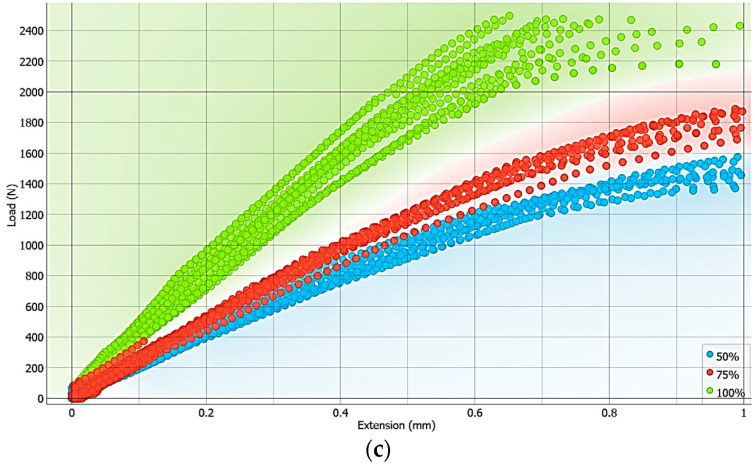
The dependency between Load and Extension: (**a**) under different heat-treated conditions, (**b**) based on layer thickness (mm), and (**c**) based on infill density.

The dependency between the applied force and the material elongation is influenced by different conditions: the way the material is heat-treated can alter its microstructure, affecting its strength, ductility, and toughness; layer thickness can significantly impact the mechanical properties; and the amount of material used within the 3D-printed object can affect the weight and strength of the final product, as highlighted in Figure 6.

#### 4.2.3. Data Preprocessing and Model Selection

For the implementation of the prediction algorithms, using machine learning, the dataset consisting of 2946 records was divided into two:

- Training set (2357 records—80%);
- Test set (589 records—20%).

The subsequent step involved configuring, implementing, and training various machine learning algorithms, followed by selecting the one with the highest score. Table 5 displays the metrics used to assess the performance of these algorithms, which are organized based on the determined score derived from comparing the five metrics.

**Figure 7:**
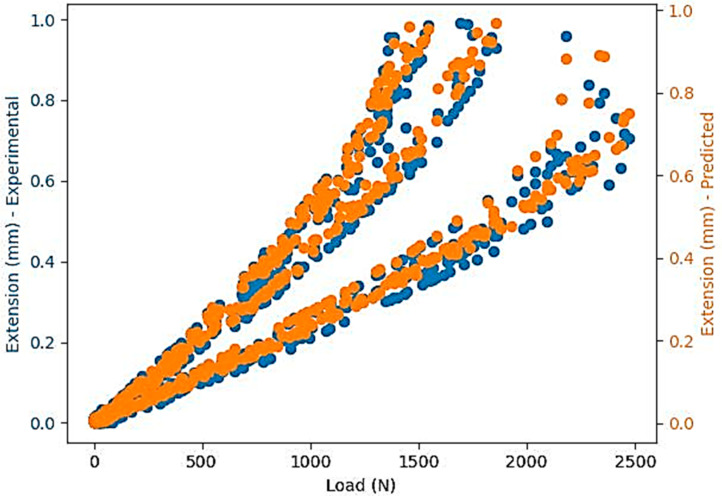
Comparison between the values in the test dataset and the prediction values.

Based on Table 5, the authors decided to use the Random Forest model. Next, the validation followed by using the model to compare the experimental results in the test dataset and the predictions obtained. The authors explored different hyperparameters of the Random Forest model to optimize its performance. To prevent the model from being dependent on a particular dataset, cross-validation techniques were used. Further visualizations were generated to demonstrate the connections between variables and their influence on predictions (Figure 7).

**Figure 8:**
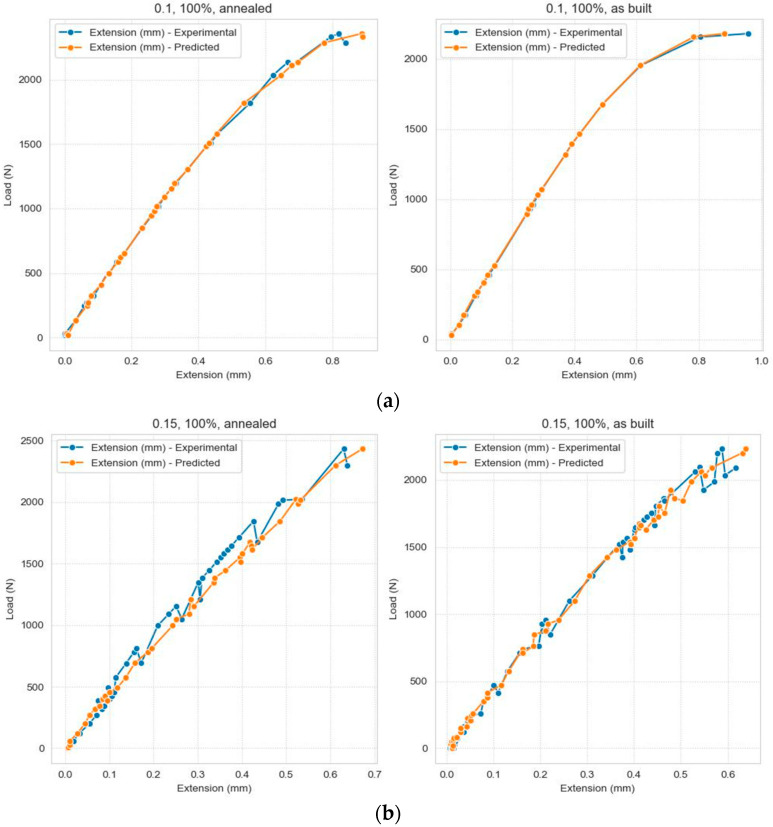

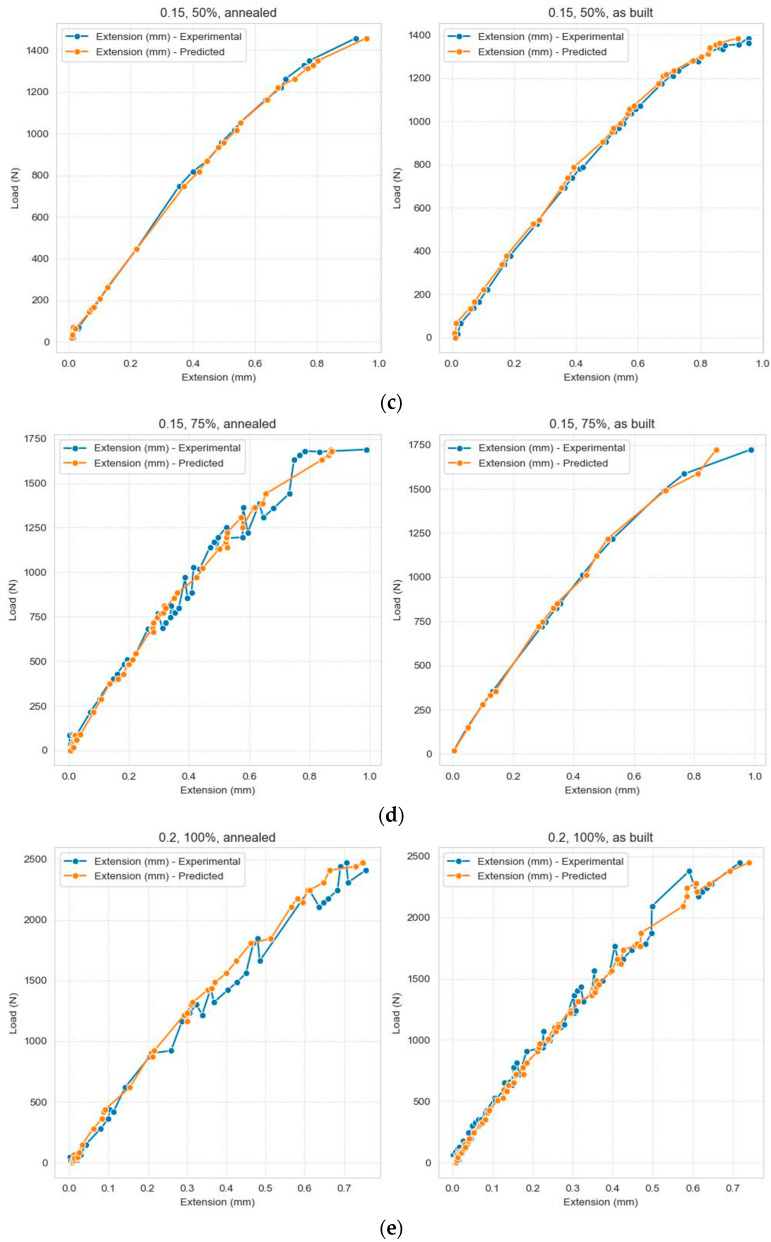

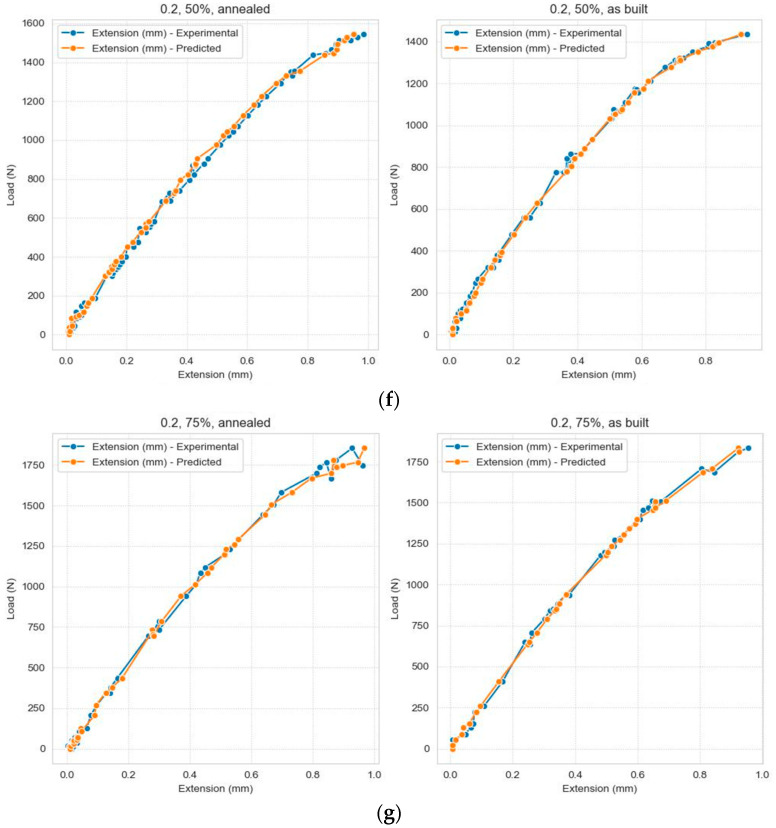
Comparison between the values in the test dataset and the predicted values, grouped according to the three categorical data: (**a**) Layer Thickness = 0.1 mm, Infill density = 100%; (**b**) Layer Thickness = 0.15 mm, Infill density = 100%; (**c**) Layer Thickness = 0.15 mm, Infill density = 50%; (**d**) Layer Thickness = 0.15 mm, Infill density = 75%; (**e**) Layer Thickness = 0.2 mm, Infill density = 100%; (**f**) Layer Thickness = 0.2 mm, Infill density = 50%; (**g**) Layer Thickness = 0.2 mm, Infill density = 75%.

To analyze the efficiency of the prediction mode, a detailed comparison was made between the dataset values and the prediction values to identify how the model performs. Figure 8 presents several scatter plots to illustrate the relationship between the actual and predicted values grouped by layer thickness, infill density, and samples.

**Figure 9:**
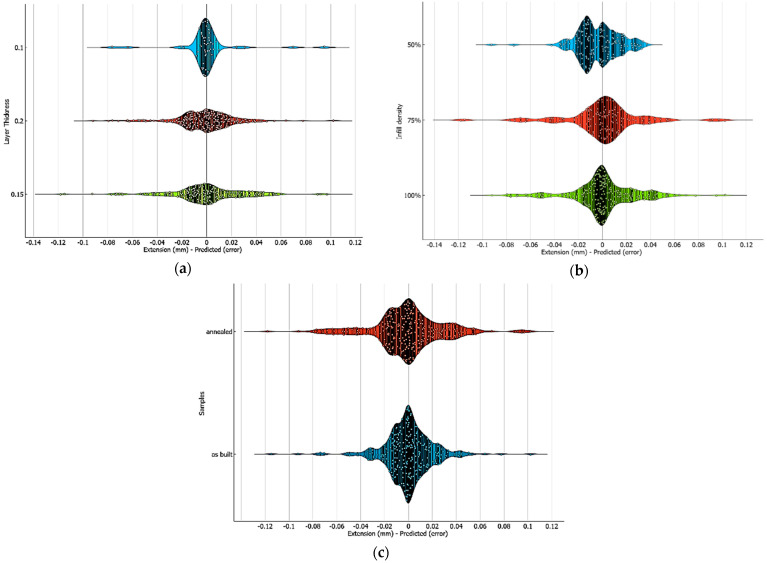
Violin plots for the prediction error distribution based on (**a**) layer thickness, (**b**) infill density, and (**c**) samples.

A classification of errors by magnitude (small errors: predictions closely match the test data. Medium errors: the deviation is still within an acceptable range. Large errors: significant discrepancies) was made with violin plots. In this paper, we utilized violin plots to visualize the distribution of prediction errors based on three key factors: layer thickness, infill density, and samples. This graphical representation allows an understanding not only of the central tendency of the error values but also of their variability and distribution. The violin’s shape in Figure 9 reflects the density of the error values across various categories. Broader sections indicate a higher concentration of errors, whereas narrower sections suggest less variability.

As can be seen from Figure 9, the predictions were not always correct. The differences between the real and predicted values can be given by several factors, such as the correlation between the chosen algorithm and the data and the number of distinct data for each test. Missing values, outliers, and noise can significantly impact the performance of predictive models.

**Figure 10:**
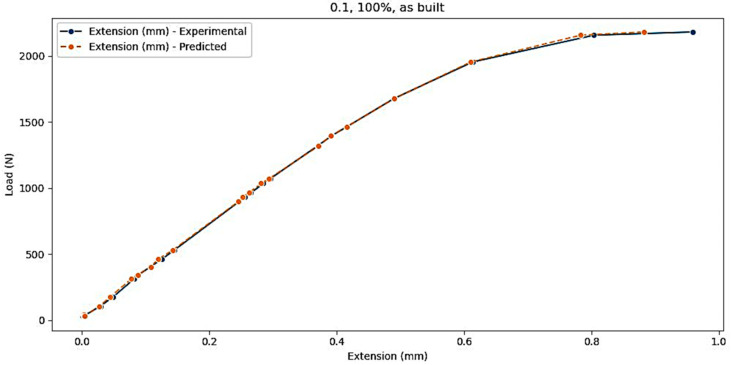
Comparison between the dataset and predicted values.

**Figure 11:**
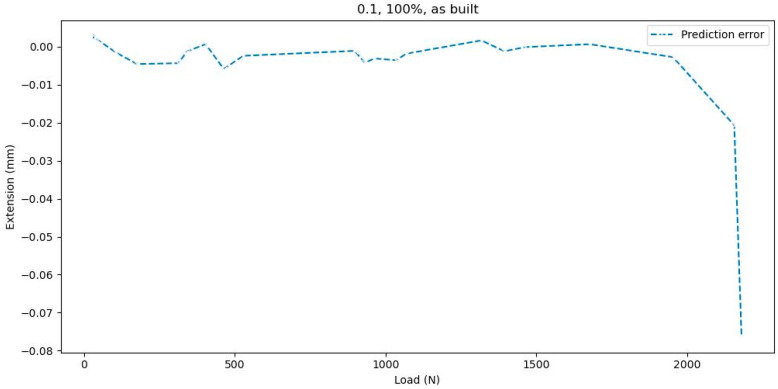
Evolution of the prediction error for the test dataset (layer thickness (mm) = 0.1; infill density = ‘100%’; and samples = ‘as built’).

The visualization of errors can be carried out in more detail if a specific experiment is extracted from the test dataset. In this case, it is considered a test where layer thickness (mm) = 0.1, infill density = ‘100%’, and samples = ‘as built’, and Figure 10 presents the comparison between the dataset and predicted values, while Figure 11 shows the evolution of the prediction error.

**Figure 12:**
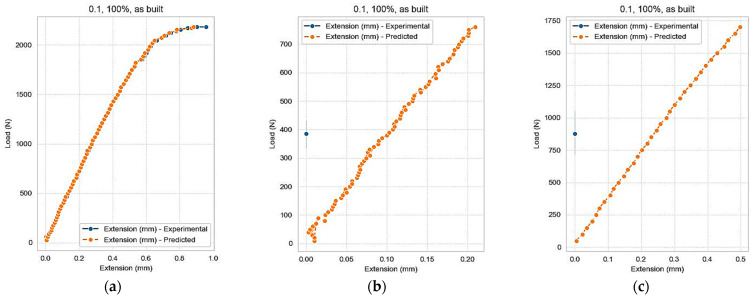
Predicted Extension: (**a**) test 1.1, (**b**) test 1.2, and (**c**) test 1.3.

To validate the prediction results, three tests were performed, as seen in Table 6 and Figure 12. The first test is the reference test and contains experimental data, while for tests 1.2 and 1.3, the values for the Load variable were modified. The value for layer thickness is 0.1 mm, infill density is set to ‘100%’, and samples = ‘as built’.

For test 1.2 and test 1.3, the Extension variable does not appear in the graph because it is set to 0 (no tests have been performed).

**Figure 13:**
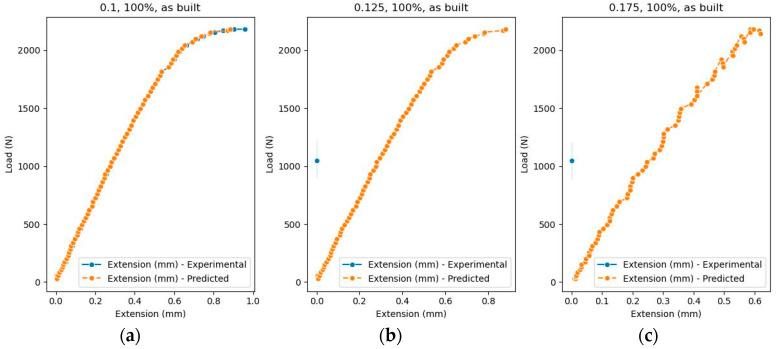
Predicted Extension: (**a**) test 2.1, (**b**) test 2.2, and (**c**) test 2.3.

Next, a new set of prediction tests was performed for values that were not found in the initial dataset, this time changing the values for layer thickness and keeping constant the values for infill density = ‘100%’ and samples = ‘as built’. The layer thickness was changed in the second test to 0.125 mm and in the third test to 0.175 mm. The values for Load were identical for the three attempts. Same as in tests 1.2 and 1.3, for the second (2.2) and third test (2.3), the Extension variable does not appear in the graph because it is set to zero, as illustrated in Figure 13.

After analyzing the performed tests, it can be specified that the predictive model works correctly. The algorithm can generate predictions for new scenarios, in which there are data that were not included in the initial dataset. The use of these predictions aims to decrease the number of experiments and ocus on the situations that are essential. By relying on accurate predictions, researchers can allocate resources more effectively, reducing the time and costs associated with unnecessary experiments.

### 4.3. Statistical Analysis

#### 4.3.1. Single-Response Analysis

**Figure 14:**
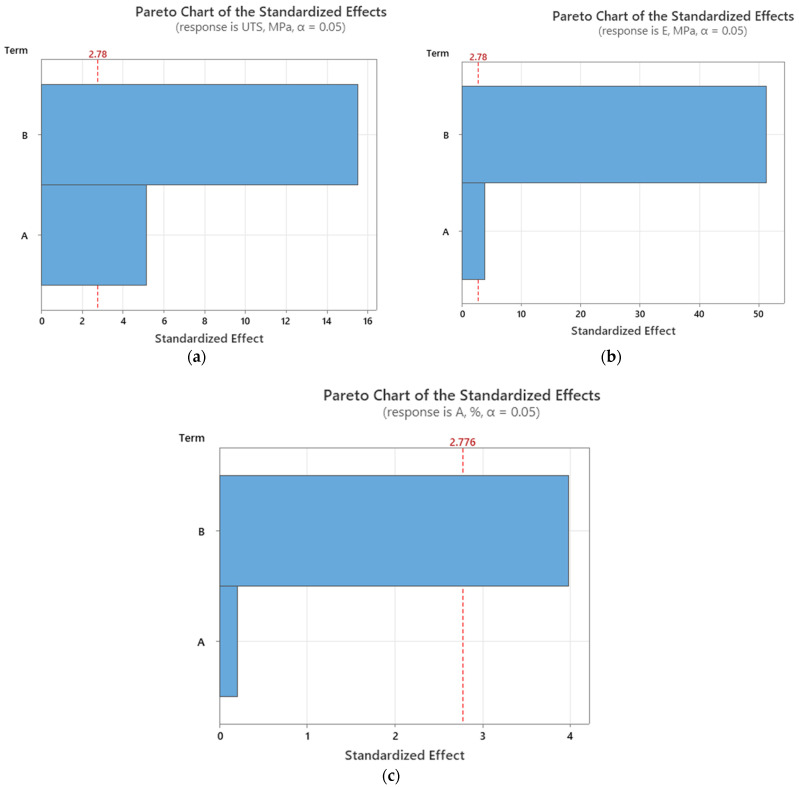
Pareto charts (as-built samples) for (**a**) ultimate tensile strength; (**b**) Young’s modulus; and (**c**) elongation at break. (Terms: A—layer thickness; B—infill percentage).

**Figure 15:**
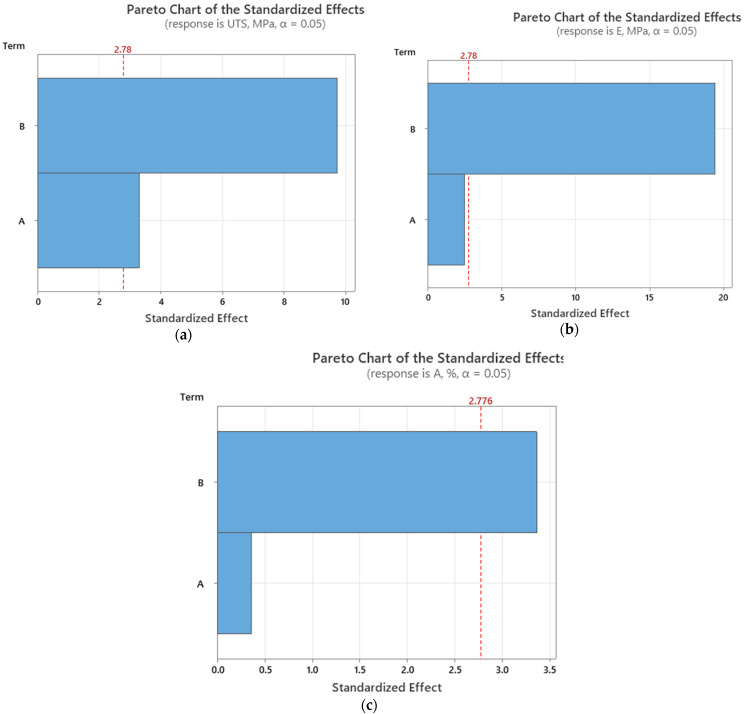
Pareto charts (annealed samples) for (**a**) ultimate tensile strength; (**b**) Young’s modulus; and (**c**) elongation at break. (Terms: A—layer thickness; B—infill percentage).

**Figure 16:**
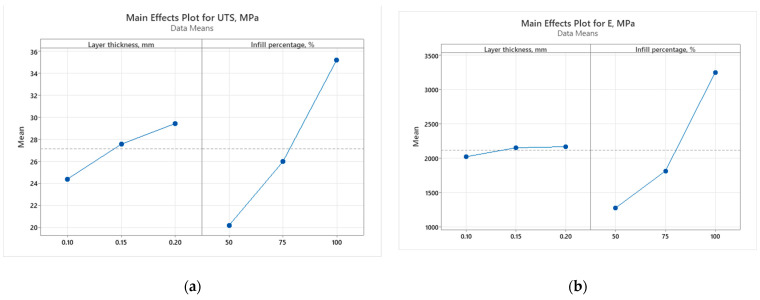

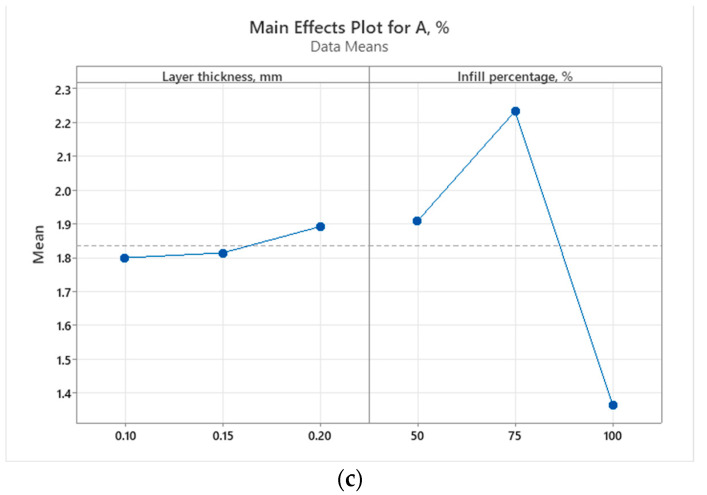
Main effect plots (as-built samples) for (**a**) ultimate tensile strength; (**b**) Young’s modulus; and (**c**) elongation at break.

**Figure 17:**
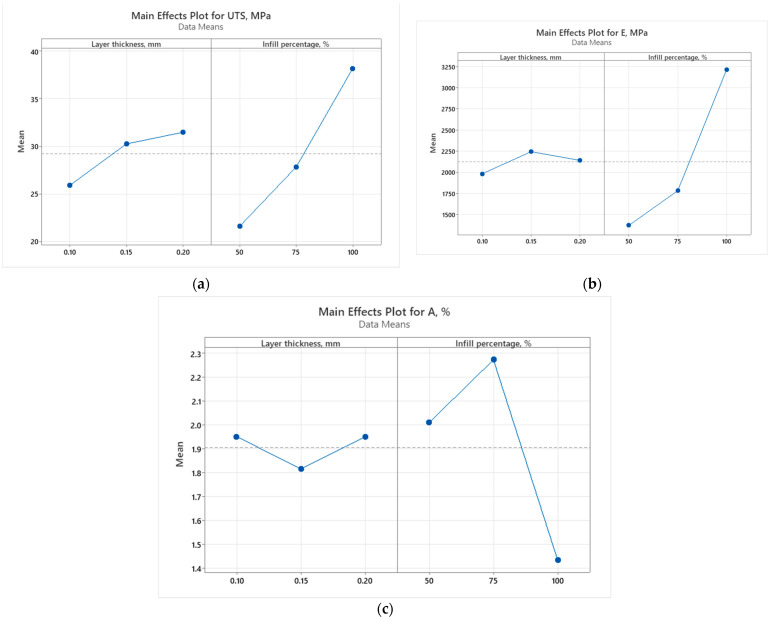
Main effect plots (annealed samples) for (**a**) ultimate tensile strength; (**b**) Young’s modulus; and (**c**) elongation at break.

To evaluate the influence of printing parameters such as infill percentage and layer thickness on the mechanical properties, graphical representations including Pareto charts (Figure 14 and Figure 15) and main effect plots (Figure 16 and Figure 17) are presented.

The Pareto charts in Figure 14 illustrate the effects of layer thickness and infill percentage on the mechanical properties of the 3D-printed PLA recycled samples.

In Figure 14a, depicting ultimate tensile strength, it is clear that the infill percentage plays a dominant role. As infill percentage increases, there is a notable improvement in tensile strength, suggesting that the internal structure of the material is significantly reinforced by a higher infill. In contrast, layer thickness shows a less pronounced effect, indicating that changes in this parameter do not have a substantial impact on the tensile strength.

The second chart (Figure 14b), which represents Young’s modulus, further emphasizes the influence of infill percentage. A higher infill percentage leads to a considerable increase in stiffness, showing that denser prints result in more rigid materials. Meanwhile, the effect of layer thickness remains less significant, suggesting that this factor contributes little to the material’s stiffness.

The chart from Figure 14c highlights the relationship between these parameters and elongation at break. Once again, infill percentage emerges as the key factor, with higher values resulting in reduced elongation, indicating that more rigid structures are less prone to stretch before breaking. Layer thickness has only a marginal influence on this property.

For the annealed samples, the Pareto chart in Figure 15a, representing ultimate tensile strength, shows that infill percentage continues to have the greatest influence on tensile strength, as it did with the as-built samples. However, its effect appears slightly reduced compared to the as-built samples, suggesting that annealing may mitigate the extent to which infill percentage dictates strength. Layer thickness still plays a minor role, reinforcing the trend observed previously, but its impact is slightly more noticeable in the annealed condition.

The second chart (Figure 15b), which focuses on Young’s modulus, demonstrates a similar pattern. The influence of infill percentage remains dominant, with annealed samples showing an even greater dependence on infill for stiffness. This suggests that annealing enhances the stiffening effect of higher infill densities. The contribution of layer thickness, though still secondary, is more apparent in the annealed condition than in the as-built samples, indicating that layer thickness may become more relevant post-annealing when considering stiffness.

In the third chart from Figure 15, which illustrates elongation at break, the trend for the annealed samples aligns with that of the as-built ones, where infill percentage plays the central role. Higher infills continue to reduce the material’s ability to stretch before breaking. However, the gap between the two parameters (infill and layer thickness) narrows in the annealed samples, suggesting that annealing may make layer thickness a more significant factor in controlling elongation.

The main effect plots for the UTS of 3D-printed recycled PLA show noticeable trends. For the as-built samples, the UTS increases slightly with an increase in layer thickness, while the infill percentage has a much more significant impact. Particularly, the UTS sees a sharp rise when moving from 50% to 100% infill, indicating that a denser infill in structures substantially enhances tensile strength. In the case of the annealed samples, a similar pattern is observed with layer thickness, where the UTS improves gradually as the thickness increases. However, the effect of infill percentage is even more pronounced compared to the as-built samples. The UTS experiences a strong boost with higher infill percentages, particularly at 100% infill, reflecting the additional strengthening due to annealing.

The graph from Figure 16b shows that in the as-built samples, Young’s modulus shows relatively little sensitivity to changes in layer thickness, with only a slight increase as the thickness grows. However, the infill percentage once again plays a major role, with a steep rise in the modulus from 50% to 100% infill, indicating a stiffer material at higher densities.

For the annealed samples, the pattern is consistent, though the overall stiffness values are lower than their as-built counterparts. There is a more gradual increase with thickness, but the infill percentage remains the primary factor in increasing Young’s modulus, particularly when the infill reaches 100%.

Elongation at break behaves differently from the other properties. In the as-built samples, elongation increases slightly with layer thickness but presents a peak at 75% infill before dropping sharply at 100%. This means that beyond a certain infill density, the material becomes more brittle. For the annealed samples, elongation at break remains relatively stable across different layer thicknesses, but a similar trend to the as-built samples is observed with infill percentage. There is a peak at 75% infill, after which the elongation drops off at 100%, reinforcing the idea that high infill densities reduce flexibility, especially after annealing.

Overall, the annealed samples exhibited higher UTS and Young’s modulus values compared to the as-built samples, particularly at higher infill percentages. However, they showed a slightly lower elongation at break, reflecting the compromise between strength and flexibility in the annealing process.

#### 4.3.2. Multi-Response Optimization

An optimization analysis was carried out to find the best combination of printing parameters to achieve maximum mechanical performance, as illustrated in Table 7 and Table 8.

Table 9 shows the ranks allocated to different options related to the printing parameters.

**Figure 18:**
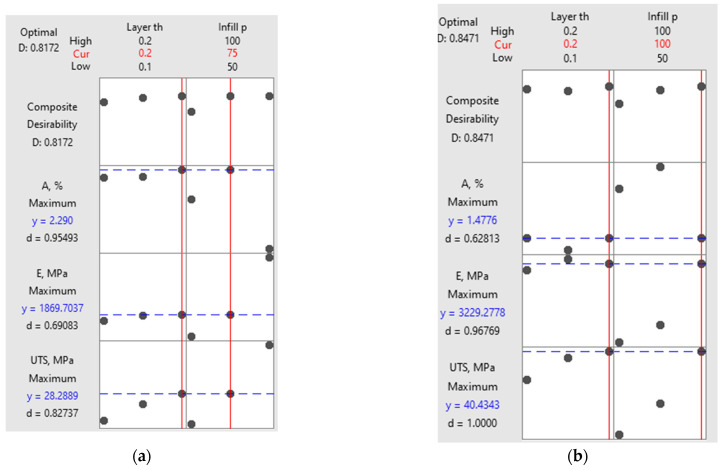
Optimization plot: (**a**) as-built samples; (**b**) annealed sample.

The optimization plot in Figure 18 illustrates the influence of each factor (columns) on the responses or composite desirability (rows). Vertical red lines indicate the current settings for each factor, while the red numbers at the top of each column represent the corresponding factor levels. Horizontal blue lines, along with their associated values, highlight the responses linked to the current factor settings.

For the as-built samples (Figure 18a), the optimal settings for achieving a high composite desirability are a layer thickness of 0.2 mm and an infill percentage of 75%. At these values, the material reaches its peak elongation at break (A%) of 2.29% and a Young’s modulus of 1869.7 MPa. However, the UTS reaches a maximum of 28.29 Mpa, slightly lower than that of the annealed samples. The composite desirability of 0.8172 indicates a good balance between strength and flexibility.

In contrast, the annealed samples (Figure 18b) show a higher composite desirability of 0.8471, reflecting improved mechanical properties after annealing. The optimization reveals that the maximum UTS jumps significantly to 40.43 Mpa, while Young’s modulus increases to 3229.3 Mpa, demonstrating the stiffening effect of annealing. However, elongation at break drops to 1.48%, which suggests that annealing improves strength and stiffness, but at the cost of reduced flexibility. The optimal settings for the annealed samples remain corresponding to a 0.2 mm layer thickness and 100% infill.

### 4.4. DSC Results

**Figure 19:**
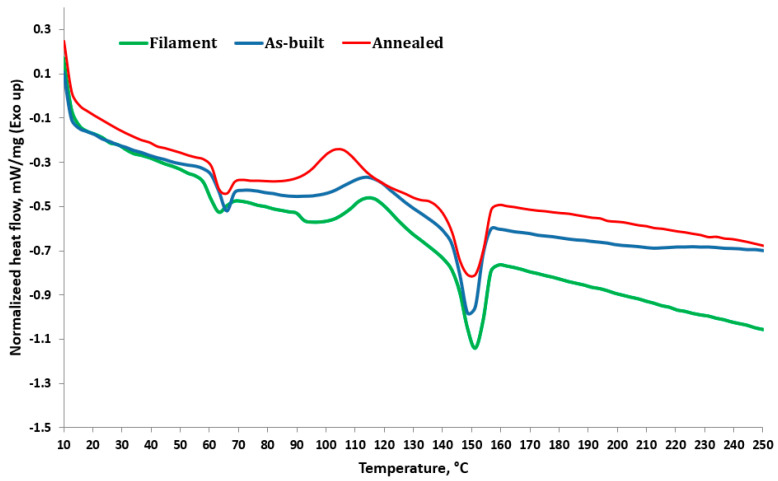
DSC curves for filament, as-built, and annealed 3D-printed samples (first heating scan).

Figure 19 and Table 10 display the samples’ thermal properties as determined by DSC analysis. Three samples of each category (as built and annealed) were examined, and it was observed that the material exhibited three primary phase transitions, denoting the glass transition material temperature (*T_g_*), cold crystallization temperature (*T_c_*), and melting temperature point (*T_m_*).

The first portion of the curve, beginning at about 60 °C, exhibits a minor shift in the baseline, which corresponds to the glass transition temperature (*T_g_*).

The *T_g_* appears to occur around the same temperature for both the as-built and annealed samples (about 60 °C–70 °C), while the annealed sample has a somewhat more pronounced endothermic step, implying that the annealing temperature causes more chain relaxation [39].

The temperature range of 90 °C to 130 °C is most likely associated with cold crystallization (exothermic peak), in which the PLA polymer begins to reorganize into a crystalline form.

The as-built sample has a stronger exothermic peak in this region, showing that the material remains mostly amorphous and crystallizes when heated. In comparison, the annealed sample has a smaller exothermic peak, indicating that the material has already crystallized during annealing [40].

Between 150 °C and 170 °C, an endothermic peak appears, which corresponds to the melting of the PLA’s crystalline areas. The annealed sample has a slightly higher melting peak, indicating that annealing increased crystallinity.

The increase in the degree of crystallinity occurs from 42.4% to 43.6%, which agrees with the study of James Finnerty et al. [41], which stated that when the recycling rate increased, the degree of crystallinity in PLA increased.

### 4.5. XRD Results

**Figure 20:**
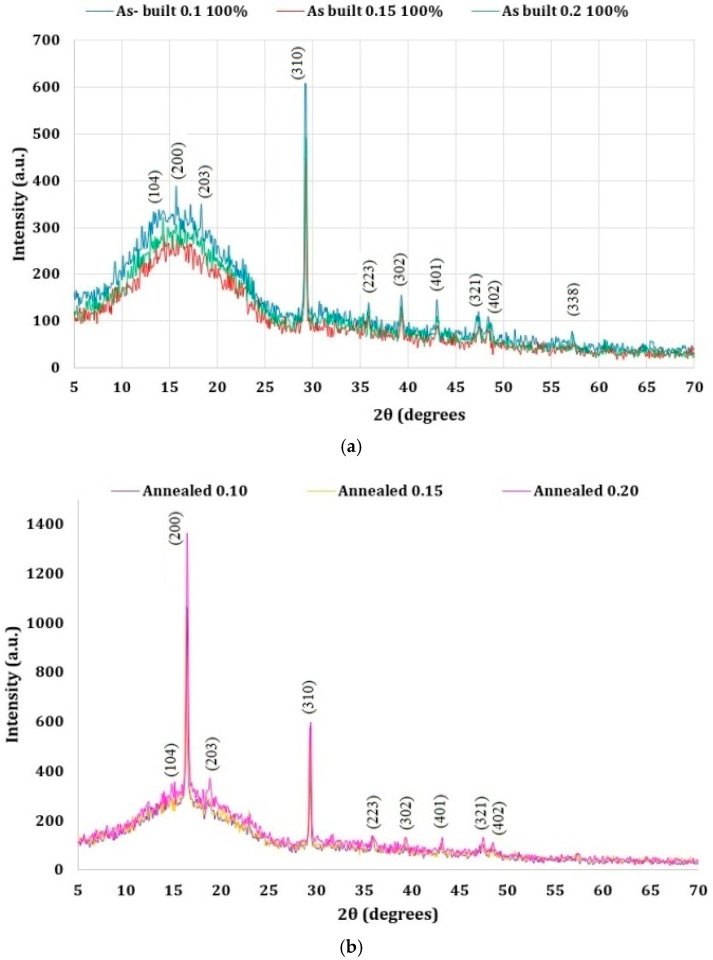
XRD patterns of PLA samples: (**a**) as built; (**b**) annealed.

Comparing the X-ray diffraction spectra recorded for the two types of PLA samples (as built and annealed), major differences can be observed related to the shape, intensity, and position of the main peaks in terms of their impact on the degree of crystallinity (Figure 20).

Using the ICDD 00-064-1624 datasheet, the PLA phase was confirmed in both types of PLA samples investigated (PLA as built and PLA annealed).

In the XRD spectra (Figure 20) of both types of PLA samples investigated, the main reflections/peaks observed (2θ degrees) were as follows: 14.80 (104), 19.02 (203), 29.25 (310), 35.80 (223), 39.30 (302), 43.10 (401), 47.40 (321), 48.35 (402), 57.20 (338). These diffraction peaks correspond to the PLA α-phase belonging to the orthorhombic system.

The XRD spectra of the as-built PLA samples showed a broad peak in the range of 10–25° (2θ), the main diffraction peak of maximum intensity (i.e., 16.62 (200)) of PLA structure not being well defined, indicating a lower crystallinity.

At the same time, the XRD spectra of the annealed PLA showed a well-defined and sharp main diffraction peak. The PLA structure’s peak was of maximum intensity, indicating a higher degree of crystallinity.

The lattice parameters of the PLA structure (α-phase) were refined using the Rietveld method and the Topas 4.1 program, assuming that the orthorhombic unit cell of our samples was similar to that published by P. De Santis [42]. The results of Rietveld refinements were in good agreement with those previous reported in the literature [30,31,43,44,45].

The values of the degree of crystallinity calculated from the X-ray spectra from Table 11 were in good agreement with the values yielded by DSC. Significant differences were observed in the XRD spectra when comparing the as-built and annealed PLA samples. After annealing, the (200) reflection significantly increased in intensity, pointing out an increase in the degree of crystallinity.

The increase in the degree of crystallinity of the PLA annealed samples is explained by the influence of the annealing treatment on the structural parameters improving the PLA microstructure by increasing its crystallization rate. PLA annealing at temperatures above the *T_g_* led to high crystallinity and good mechanical properties, *T_g_* values higher than the processing temperatures not allowing chain orientation and crystallization after cooling. Previous studies [40,41,46,47] reported an even greater increase in the degree of crystallinity, mechanical properties, and heat deflection temperature of PLA by combining the annealing with nucleation.

## 5. Conclusions

The performed investigation was conducted to compare the performance of recycled PLA 3D-printed parts with virgin PLA material and to analyze the influence of annealing heat treatment on the tensile properties of recycled 3D-printed samples. It was found that the as-built samples showed a higher UTS and Young’s modulus with greater infill densities and thicker layers but lower ductility. Annealing improved mechanical strength and stiffness in most cases, while its effect on ductility was minimal or slightly positive, suggesting that annealing could be a beneficial post-processing step for recycled PLA to enhance its mechanical performance.

Both the DSC and XRD analyses revealed a slight but noticeable increase in crystallinity following the annealing process. Specifically, the DSC results showed crystallinity rising from 42.4% in the as-built state to 43.6% after annealing. Additionally, XRD analysis provided further evidence of increased crystallinity. Crystallinity increased across all layer thicknesses following annealing: at 0.10 mm, it rose from 35.77% in the as-built state to 43.09%; at 0.15 mm, from 32.44% to 43.45%; and at 0.20 mm, from 34.55% to 45.86%. These findings, although modest, suggest that the selected annealing temperature and time had a positive effect on the crystallinity of recycled PLA.

In summary, annealing enhances certain mechanical properties, particularly stiffness, and slightly balances the influence of layer thickness alongside infill percentage, while maintaining the general trends observed in the as-built samples.

The multi-objective optimization based on desirability analysis allowed the establishment of a combination of input parameters (layer thickness and infill percentage) determining maximum tensile properties. For the as-built samples, the optimal settings were a layer thickness of 0.2 mm and a 75% infill, while the optimal settings for the annealed samples were a 0.2 mm layer thickness and a 100% infill.

Thicker layers (0.2 mm) contributed to stronger tensile properties, enabling recycled PLA to match or even surpass the strength of virgin PLA, particularly when infill percentages were high. Thinner layers (0.1 mm), however, resulted in weaker bonds between the layers, causing recycled PLA to exhibit reduced tensile strength compared to virgin PLA, especially at lower infill percentages.

The results showed that recycled PLA can perform nearly as well as virgin PLA, provided that thicker layers and higher infill percentages are used. The optimal settings differ between the as-built and annealed samples, with annealing favoring a higher infill percentage to maximize strength and stiffness. Annealing enhanced tensile strength and stiffness, as shown by the higher UTS and Young’s modulus values of the annealed samples. However, this comes at the expense of flexibility, as indicated by their lower elongation at break compared to the as-built samples. Therefore, the results of this paper strongly evidence the necessity of annealing recycled PLA material above the glass transition temperature in order to ensure a high degree of crystallinity and good mechanical properties.

The ability to retain or enhance tensile strength by adjusting layer thickness and infill percentage opens up a wide range of applications for recycled materials. With careful optimization, industries can integrate recycled PLA into functional parts, reducing the reliance on virgin plastics and supporting a more sustainable manufacturing process.

A limitation of this study is that it focused on a specific type of recycled PLA, without systematically exploring variations in recycling sources, annealing times, temperatures, or controlled degradation. While the mechanical properties of recycled PLA were improved, the gains were modest compared to virgin PLA. Future research should investigate the impact of these additional factors and assess the long-term performance of recycled PLA to better understand its potential as a sustainable alternative in various industrial applications.

## Figures and Tables

**Table 1:** The printing settings.

Printing Options	Value
Shell width (mm)	1
Infill speed (mm/s)	70
Estimated print time (min)	46
Estimated filament used (g)	10.60
Extruder temperature (°C)	210
Bed temperature (°C)	60
Platform addition	Raft only

**Table 2:** Parameters and corresponding levels utilized in the DOE analysis.

Parameter	Level		
	1	2	3
Layer thickness, mm	0.10	0.15	0.20
Infill percentage, %	50	75	100

**Table 3:** Types of data used for dataset.

Name	Type	Role	Values
Layer Thickness	Categorical	Feature	0.1, 0.15, 0.2
Infill density	Categorical	Feature	50%, 75%, 100%
Samples	Categorical	Feature	Annealed, as built
Load	Numeric	Feature	
Extension	Numeric	Target	

**Table 4:** Statistical representations of the dataset.

Statistics	Layer Thickness	Load	Extension
count	2946	2946	2946
mean	0.169756	848.5495	0.318202
std	0.033804	672.5881	0.269695
min	0.1	0	0
25%	0.15	214	0.068647
50%	0.2	768.94	0.26532
75%	0.2	1357.6	0.517402
max	0.2	2496.7	0.99793

**Table 5:** Key metrics used to evaluate the performance of ML algorithms.

Model	MSE	RMSE	MAE	MdAPE	R^2^
Random Forest	0.001	0.027	0.018	6.059579664	0.990
Gradient Boosting	0.001	0.028	0.019	6.698080301	0.989
Tree	0.001	0.030	0.020	6.977544282	0.988
AdaBoost	0.001	0.031	0.020	6.950282399	0.987
Neural Network	0.001	0.032	0.021	6.224877491	0.986
Linear Regression	0.008	0.090	0.073	21.99528675	0.889
SVM Learner	0.012	0.108	0.085	17.17770959	0.841
kNN	0.015	0.124	0.082	26.72113087	0.788

**Table 6:** First and last 5 values for Load and predicted Extension for the 3 tests.

Prediction Test 1.1		Prediction Test 1.2		Prediction Test 1.3	
Load (N)	Extension (mm)—Predicted	Load (N)	Extension (mm)—Predicted	Load (N)	Extension (mm)—Predicted
32.525	0.0035286	10	0.0098069	50	0.00488235
44.439	0.00324737	20	0.0101538	100	0.023429
59.47	0.00423718	30	0.00729629	150	0.0363373
40.525	0.00278179	40	0.00278179	200	0.0537019
31.655	0.00434033	50	0.00488235	250	0.0653139
…					
2141.1	0.779581	720	0.194466	1500	0.428727
2156.1	0.783324	730	0.200634	1550	0.449607
2170.2	0.870527	740	0.201108	1600	0.461231
2182.2	0.882195	750	0.201739	1650	0.482956
2180.4	0.882195	760	0.209748	1700	0.497015

**Table 7:** Optimization goals for mechanical properties of as-built samples.

Response	Goal	Lower	Target	Upper	Weight	Importance
A, %	Maximum	1.18	2.41		0.33	1
E, MPa	Maximum	1201.50	3441.50		0.33	1
UTS, MPa	Maximum	16.36	39.42		0.33	1

**Table 8:** Optimization goals for mechanical properties of annealed samples.

Response	Goal	Lower	Target	Upper	Weight	Importance
A, %	Maximum	1.22	2.45		0.33	1
E, MPa	Maximum	1163.50	3329.67		0.33	1
UTS, MPa	Maximum	16.79	37.21		0.33	1

**Table 9:** Composite desirability and ranks.

		As Built		Annealed	
Layer Thickness, mm	Infill Percentage, %	Composite Desirability	Rank	Composite Desirability	Rank
0.10	50	0.385318	9	0.458268	9
0.10	75	0.740012	5	0.739367	6
0.10	100	0.735175	6	0.819881	2
0.15	50	0.580915	8	0.655372	7
0.15	75	0.792769	3	0.804551	4
0.15	100	0.766233	4	0.797615	5
0.20	50	0.620928	7	0.648887	8
0.20	75	0.817232	1 *	0.809841	3
0.20	100	0.817221	2	0.847086	1 *

* The highlighted line corresponds to the optimal values (having Rank 1) of the printing settings.

**Table 10:** Thermal characteristics of the analyzed samples.

Sample Type	Mass, mg	*T_g_*, °C	*T_c_*, °C	*T_m_*, °C	*X_C_*, %
Filament	3.43	63.30 ± 0.12	114.00 ± 0.27	151.30 ± 0.22	-
As built	3.90	65.60 ± 0.14	113.95 ± 0.23	149.67 ± 0.17	42.4
Annealed	3.78	64.78 ± 0.21	104.63 ± 0.15	149.87 ± 0.19	43.6

**Table 11:** Degree of crystallinity for the investigated 3D-printed PLA samples.

Layer Thickness	XRD *X_C_* (%)	
	PLA As Built	PLA Annealed
0.10	35.77	43.09
0.15	32.44	43.45
0.20	34.55	45.86

## Data Availability

Data are contained within the article.
